# Cannabidiol (CBD) Is a Novel Inhibitor for Exosome and Microvesicle (EMV) Release in Cancer

**DOI:** 10.3389/fphar.2018.00889

**Published:** 2018-08-13

**Authors:** Uchini S. Kosgodage, Rhys Mould, Aine B. Henley, Alistair V. Nunn, Geoffrey W. Guy, E. L. Thomas, Jameel M. Inal, Jimmy D. Bell, Sigrun Lange

**Affiliations:** ^1^Cellular and Molecular Immunology Research Centre, School of Human Sciences, London Metropolitan University, London, United Kingdom; ^2^Research Centre for Optimal Health, Department of Life Sciences, University of Westminster, London, United Kingdom; ^3^GW Research, Sovereign House Vision Park, Cambridge, United Kingdom; ^4^School of Life and Medical Sciences, University of Hertfordshire, Hatfield, United Kingdom; ^5^Tissue Architecture and Regeneration Research Group, Department of Biomedical Sciences, University of Westminster, London, United Kingdom; ^6^Department of Pharmacology, University College London School of Pharmacy, London, United Kingdom

**Keywords:** exosomes, microvesicles (MVs), cannabidiol (CBD), peptidylarginine deiminase (PAD), cancer, inflammation, mitochondria, combinatory treatment

## Abstract

Exosomes and microvesicles (EMV) are lipid bilayer-enclosed structures, released by cells and involved in intercellular communication through transfer of proteins and genetic material. EMV release is also associated with various pathologies, including cancer, where increased EMV release is amongst other associated with chemo-resistance and active transfer of pro-oncogenic factors. Recent studies show that EMV-inhibiting agents can sensitize cancer cells to chemotherapeutic agents and reduce cancer growth *in vivo*. Cannabidiol (CBD), a phytocannabinoid derived from *Cannabis sativa*, has anti-inflammatory and anti-oxidant properties, and displays anti-proliferative activity. Here we report a novel role for CBD as a potent inhibitor of EMV release from three cancer cell lines: prostate cancer (PC3), hepatocellular carcinoma (HEPG2) and breast adenocarcinoma (MDA-MB-231). CBD significantly reduced exosome release in all three cancer cell lines, and also significantly, albeit more variably, inhibited microvesicle release. The EMV modulating effects of CBD were found to be dose dependent (1 and 5 μM) and cancer cell type specific. Moreover, we provide evidence that this may be associated with changes in mitochondrial function, including modulation of STAT3 and prohibitin expression, and that CBD can be used to sensitize cancer cells to chemotherapy. We suggest that the known anti-cancer effects of CBD may partly be due to the regulatory effects on EMV biogenesis, and thus CBD poses as a novel and safe modulator of EMV-mediated pathological events.

## Introduction

Extracellular vesicles released from cells are classified into exosomes, microvesicles and apoptotic bodies (György et al., 2011). Exosomes and microvesicles (EMVs) are lipid-bilayer structures that carry molecules characteristic of their parental cells to recipient cells, mediating intercellular communication and affecting various physiological and pathological processes including cell migration, differentiation and angiogenesis (Ansa-Addo et al., 2010; Muralidharan-Chari et al., 2010; Turola et al., 2012; Colombo et al., 2014; Batrakova and Kim, 2015; Kholia et al., 2016).

Microvesicles (MVs) are phospholipid-rich cell-membrane derived vesicles (100–1000 nm) released as part of normal physiology as well as during apoptosis or upon stimulation (Piccin et al., 2007; Inal et al., 2013). The release of MVs can be mediated via calcium ion influx through stimulation of cation channels such as the ATP-gated P2X7, through pores created by sublytic complement, or via calcium released by the endoplasmic reticulum (Turola et al., 2012; Raposo and Stoorvogel, 2013; Stratton et al., 2015a,b). This increase in cytosolic calcium results in cytoskeletal reorganization and membrane asymmetry, followed by subsequent MV blebbing (Inal et al., 2012, 2013; Kholia et al., 2015; Kosgodage et al., 2017; Tricarico et al., 2017). MV formation can also be caused by mitochondrial stress, which leads to increased membrane permeability and leakage of reactive oxygen species (ROS), cytochrome C and apoptosis inducing factor into the cytoplasm. This results in the formation of the apoptosome – which, during pseudoapoptosis, can be formed into MVs for the export of hazardous agents (Inal et al., 2013).

Exosomes (30–100 nm) are generated intracellularly as they are formed after the invagination of the endosome membrane, resulting in intraluminal vesicle formation and the appearance of multivesicular endosomes, which then release exosomes from the plasma membrane via exocytosis (Kowal et al., 2014; van Niel et al., 2018). Crucial cellular components for exosomal biogenesis are ESCRT (endosomal sorting complexes required for transport), sphingolipid ceramide, syntetin and syndecan, and tetraspanins (Théry et al., 2002; Baietti et al., 2012; Colombo et al., 2013; Costa, 2017; Hessvik and Llorente, 2018). The secretion of exosomes is also affected via purinergic receptors such as P2X7 (Qu et al., 2007), by microenvironmental pH (Federici et al., 2014) and calcium (Savina et al., 2003; Kramer-Albers et al., 2007).

Exosome and microvesicles are emerging as novel therapeutic targets in treatment of disease as they have been shown to contribute to inflammatory processes (Foers et al., 2017) and the progression of numerous pathologies including autoimmune diseases (Antwi-Baffour et al., 2010; Withrow et al., 2016; Perez-Hernandez et al., 2017), cancers (Luga et al., 2012; Jorfi et al., 2015; Kholia et al., 2015; Stratton et al., 2015a; Sung et al., 2015; Tkach and Théry, 2016; Moore et al., 2017; Sung and Weaver, 2017) and neurodegenerative diseases (Colombo et al., 2012; Gupta and Pulliam, 2014; Porro et al., 2015; Basso and Bonetto, 2016). In cancer patients, elevated EMV levels have for example been demonstrated in the blood (Ginestra et al., 1998; Kim et al., 2003; Zwicker et al., 2009) and EMVs can also aid tumor spread and survival as they transport various micro RNAs, pathological growth factor receptors and soluble proteins (Muralidharan-Chari et al., 2010; Inal et al., 2012; Hoshino et al., 2015). Circulating EMVs in various body fluids such as cerebrospinal fluid, urine and blood, may in addition serve as reliable biomarkers of pathophysiological processes (Piccin et al., 2007; Inal et al., 2012, 2013; Porro et al., 2015). Besides contributing to disease pathology, EMVs are being considered as therapeutic vehicles themselves (György et al., 2015; Moore et al., 2017).

It has been shown that EMV shedding from cancer cells aids increased active drug efflux and thus contributes to their resistance to chemotherapeutic agents (Bebawy et al., 2009; Tang et al., 2012; Jorfi and Inal, 2013; Pascucci et al., 2014; Jorfi et al., 2015; Saari et al., 2015; Soekmadji and Nelson, 2015; Aubertin et al., 2016; Koch et al., 2016; Muralidharan-Chari et al., 2016). Recent studies on pharmacological inhibition of EMV release have shown that such interventions could be a new strategy to render cancer cells more susceptible to anticancer drug treatment (Tang et al., 2012; Federici et al., 2014; Jorfi et al., 2015; Koch et al., 2016; Muralidharan-Chari et al., 2016; Kosgodage et al., 2017). Such approaches have recently been shown to be effective *in vivo*, demonstrating that the application of EMV inhibitors can effectively sensitize tumors to chemotherapy (Jorfi et al., 2015; Koch et al., 2016; Muralidharan-Chari et al., 2016), reduce drug efflux (Federici et al., 2014; Koch et al., 2016; Muralidharan-Chari et al., 2016) and reduce the dose of anti-cancer drug required to limit tumor growth *in vivo* (Jorfi et al., 2015). Pharmacological non-toxic agents that can selectively manipulate extracellular vesicle release may thus be relevant not only to cancer but also to other pathologies involving EMV release (Lange et al., 2017).

Cannabidiol (CBD) (Mechoulam et al., 2002), a phytocannabinoid derived from *Cannabis sativa*, is anxiolytic (Blessing et al., 2015) and has analgesic, anti-inflammatory, antineoplastic and chemo-preventive activities (Martin-Moreno et al., 2011; Pisanti et al., 2017). CBD has been shown to have a plethora of molecular targets, including the classical endocannabinoid system, while effects that do not involve the classical cannabinoid system are also gaining increased attention (Ibeas Bih et al., 2015; Pisanti et al., 2017). CBD is generally safe at therapeutic doses, shows biphasic effects on the immune system, and has demonstrated anti-cancer activity *in vivo* (Bergamaschi et al., 2011; Massi et al., 2013; Haustein et al., 2014; Velasco et al., 2016). Critically, CBD has been shown to be effective in various EMV-linked pathologies (Velasco et al., 2016), and seems to modulate mitochondrial function, including ATP, ROS and proton leak, as well as uptake and release of calcium (Ryan et al., 2009; Mato et al., 2010; Rimmerman et al., 2013; Cui et al., 2017). These observations may be relevant as mitochondria are key in modulating calcium signaling (Szabadkai and Duchen, 2008; Rizzuto et al., 2012) and importantly, altered calcium signaling and mitochondrial function are hallmarks of many cancers (Boland et al., 2013; Stefano and Kream, 2015; Monteith et al., 2017). This study therefore aimed to investigate putative modulatory effects of CBD on EMV release and to further establish whether CBD had combinatory effects with the recently described EMV-inhibitor Cl-amidine (Luo et al., 2006; Kholia et al., 2015; Kosgodage et al., 2017). For proof of principle we used three cancer cell lines, prostate cancer (PC3), hepatocellular carcinoma (HEPG2) and breast adenocarcinoma (MDA-MB-231). Here we show effects of CBD on EMV release, on mitochondrial function, as well as on STAT3 expression, which amongst other is associated with mitochondrial respiration and Ca^2+^ regulation in the mitochondrion (Wegrzyn et al., 2009; Yang et al., 2015; Yang and Rincon, 2016), alongside modulatory effects on prohibitin, a pleiotropic protein involved in cellular proliferation and mitochondrial housekeeping (Peng et al., 2015; Ande et al., 2017). Our findings suggest a new link between the emerging understanding of anti-cancer effects of CBD and its modulatory effects on EMV biogenesis in cancer cells, described here for the first time.

## Materials and Methods

### Cell Cultures

Human prostate adenocarcinoma (PC3 and ECACC), human hepatocellular carcinoma (HEPG2 and ECACC) and human breast adenocarcinoma (MDA-MB-231; a kind gift from Dr T. Kalber, UCL) cell lines were maintained at 37°C/5% CO_2_, in growth medium containing 10% EMV-free Foetal Bovine Serum (FBS) and RPMI (Sigma, United Kingdom). The cells were split every 3–5 days, depending on confluence, washed twice with EMV-free Dulbecco’s Phosphate Buffered Saline (DPBS), prepared as described before (Kosgodage et al., 2017) and detached by incubation for 10–15 min at 37^o^C with 0.25% (v/v) trypsin/EDTA, followed by two washes by centrifugation using EMV-free DPBS at 200 *g*/5 min. Before the start of every experiment, cell numbers and viability were determined by Guava ViaCount assay (Guava Millipore) and exponentially growing cells with viabilities of ≥95% were used.

### Cell Viability Assays

The Guava EasyCyte 8HT flow cytometer (Millipore) and ViaCount assay (Guava Millipore) were used to count and determine viability of cells before the start of every experiment and to assess cell viability after treatment with EMV inhibitors, as previously described (Jorfi et al., 2015; Kosgodage et al., 2017). Cell viability after cisplatin treatment (see 2.9) was assessed by MTT [3-(4,5-dimethylthiazol-2-yl)-2,5-diphenyltetrazolium bromide] assay, performed according to the manufacturer’s instructions (Sigma, United Kingdom).

### Effects on EMV Biogenesis Using CBD and Cl-Amidine

For assessment of effects of CBD and Cl-amidine on EMV generation, PC3, HEPG2 and MDA-MB-231 cells were seeded at a density of 3.8 × 10^5^ cells/well, in triplicate, in 12-well microtiter plates, using pre-warmed serum- and EMV-free RPMI 1640 (Sigma-Aldrich, United Kingdom). To ensure that the medium was EMV free, it was centrifuged at 70,000 *g*/24 h and filtered through a 0.22 μm pore size membrane before use. For testing of putative inhibitory or modulatory effects on EMV release, the cells were then incubated with CBD (1 or 5 μM), Cl-amidine (50 μM) or with a combination of CBD (5 μM) and Cl-amidine (50 μM), for 60 min at 37°C/5% CO_2_, while control cells were treated with either DMSO (0.001%) or PBS for CBD and Cl-amidine, respectively. The following concentrations of CBD (GW Pharmaceuticals, United Kingdom) were used: 1 or 5 μM in 0.001% DMSO, based on clinically relevant doses for CBD (Bergamaschi et al., 2011); while Cl-amidine (a kind gift from Prof P.R. Thompson, UMASS) was used at 50 μM concentration (in PBS) as previously determined as an optimal dose for maximum EMV inhibition in several cancer cell lines (Kholia et al., 2015; Kosgodage et al., 2017). For testing of a combinatory effect on EMV release, CBD was applied at 5 μM together with Cl-amidine at 50 μM concentrations. After the 1 h incubation period, the supernatants from each well were collected from the cell preparations, transferred to sterile 1.5 ml Eppendorf tubes (kept on ice) and centrifuged at 200 *g* for 5 min at 4°C to remove the cell debris. The resulting supernatants were kept on ice and subsequently treated for isolation of EMVs, as described below, to include both exosomes and MVs based on previously established protocols (Lötvall et al., 2014; Kholia et al., 2015; Kosgodage et al., 2017; Witwer et al., 2017).

### Isolation of EMVs

Exosome and microvesicles were isolated from the CBD, Cl-amidine, and CBD plus Cl-amidine treated cell culture supernatants, as well as from the control treated cells (DMSO or PBS), by differential centrifugation as follows: First, whole cells were removed by spinning at 200 *g*/5 min at 4°C. The supernatants were then collected and further centrifuged at 4,000 *g* for 60 min at 4°C, to remove cell debris. The resulting supernatants were thereafter collected and centrifuged again at 25,000 *g* for 1 h at 4°C. The resulting EMV pellets were collected and the supernatants were discarded. Next, the isolated EMV pellets were resuspended in sterile-filtered (0.22 μm) EMV-free Dulbecco’s PBS (DPBS) and thereafter centrifuged again at 25,000 *g* for 1 h at 4°C to remove proteins that may have bound to the EMV surface. The DPBS supernatant was thereafter discarded and the resulting isolated EMV pellets were resuspended in 200 μl of sterile EMV-free DPBS for further nanoparticle tracking analysis (NTA), using the Nanosight (LM10; Nanosight, Amesbury, United Kingdom). Each experiment was repeated three times and performed in triplicate.

### Nanoparticle Tracking Analysis (NTA, NanoSight LM10)

To determine size distribution of isolated EMVs, nanoparticle tracking analysis (NTA), based on the Brownian motion of vesicles in suspension (Soo et al., 2012), was used. A Nanosight LM10, equipped with a sCMOS camera and a 405 nm diode laser, was used to enumerate the EMVs. The NTA software 3.0 was used for data acquisition and processing according to the manufacturer’s instructions (Malvern). The ambient temperature was set at 23°C, while background extraction and automatic settings were applied for the minimum expected particle size, minimum track length and blur. For calibration, silica beads (100 nm diameter; Microspheres-Nanospheres, Cold Spring, NY) were used. The samples were diluted 1:50 in sterile-filtered, EMV-free DPBS. To maintain the number of particles in the field of view approximately in-between 20 and 40, the minimum concentration of samples was set at 5 × 10^7^ particles/ml. For capturing, the screen and camera gain were set at 8 and 13, respectively; while for processing, the settings were at nine and three for screen gain and detection threshold, respectively, as according to the manufacturer’s instructions (Malvern). Five × 30 s videos were recorded for each sample, measurements with at least 1,000 completed tracks were used for analysis and the resulting replicate histograms were averaged. Each experiment was repeated three times and performed in triplicate.

For verification of the presence of exosomes within the 30–100 nm sized vesicle peak, according to NTA analysis, and MVs within the 101–900 nm sized vesicle peak, according to NTA analysis (**Supplementary Figures 1A,B**), MVs were pelleted first, from the EMV supernatants, by centrifugation at 11,000 *g* for 30 min at 4°C, and thereafter the presence of MVs was assessed by flow cytometry for Annexin V-FITC binding as a measure of phosphatidylserine exposition characteristic for MVs (**Supplementary Figure 1C**). The remaining supernatant was further centrifuged for the isolation of the smaller sized exosomes (<100 nm) at 100,000 *g* for 1 h at 4°C, using the Beckman-Coulter Type 60 Ti rotor. Exosomes were then characterized by Western blotting for the exosome marker CD63 (**Supplementary Figure 1D**). Exosomes and MVs were also verified by transmission electron microscopy (**Supplementary Figures 1A,B**) according to previously described methods and recommendations (Ansa-Addo et al., 2010; Lötvall et al., 2014; Stratton et al., 2015a; Kosgodage et al., 2017; Witwer et al., 2017).

**FIGURE 1:**
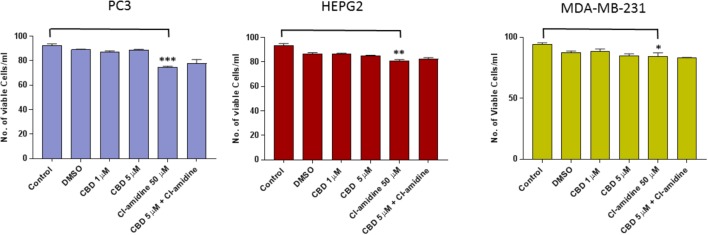
CBD does not affect cell viability of PC3, HEPG2, and MDA-MB-231 cells after 1 h treatment. The Guava EasyCyte 8HT flow cytometer (Millipore) and ViaCount assay were used to count and determine viability of CBD treated cells compared to EMV inhibitor Cl-amidine and DMSO treated control cells after 1 h incubation (^∗^*p* ≤ 0.05;^∗∗^*p* ≤ 0.01;^∗∗∗^*p* ≤ 0.001).

### Western Blotting Analysis for Changes in Exosome-Associated CD63 Expression

HEPG2, PC3, and MDA-MB231 cells were grown as a monolayer in T75 flasks (Nunc, United States) until approximately 80% confluent. The media was removed, the cells washed in DPBS and fresh medium added, containing 5 μM CBD or 0.001% DMSO as control treatment. After 1 h incubation with CBD or DMSO, respectively, the media containing EMVs was removed and first centrifuged at 4000 *g* for 30 min at 4°C for removal of cell debris. The resulting supernatant was thereafter ultra-centrifuged for 1 h at 100,000 *g* at 4°C, collecting the resulting EMV pellet, which was washed in 500 μl DPBS and subsequently ultra-centrifuged again at 100,000 *g* for 1 h at 4°C. The isolated EMV pellets were thereafter subjected to protein extraction, using 50 μl RIPA buffer (Sigma, United Kingdom; supplemented with protease inhibitor cocktail P8340, Sigma United Kingdom), per pellet, by pipetting up and down 20 times and thereafter incubating the pellets in RIPA+ buffer on a shaking platform for 1 h on ice. Thereafter, extracted proteins were isolated by centrifugation at 16,000 *g* for 20 min at 4°C, collecting the protein containing supernatant. The corresponding HEPG2, PC3, and MDA-MB231 cells were also collected from each flask for internal comparison of cell amount (as estimated by β-actin) versus vesicles released between CBD treatment and DMSO controls. Cell pellets were treated with equal amounts of RIPA+ buffer using 50 μl buffer per pelleted cells from each flask. Cell protein isolates were then prepared in the same way as EMV protein isolates. The resulting EMV and cell protein preparations were then reconstituted 1:1 in 2× Laemmli sample buffer (BioRad, United Kingdom) containing 5% β-mercaptoethanol (BioRad) and boiled at 100°C for 5 min before protein separation on 4–20% Mini-Protean TGX gels (BioRad). For each EMV sample 20 μl were loaded, while for each cell lysate preparation, 10 μl were loaded per lane. For immunoblotting, proteins were transferred to 0.45 μm nitrocellulose membranes (BioRad) using semi-dry Western blotting at 15 V constant for 1 h, even transfer was assessed using Ponceau S staining (Sigma) and the membranes were blocked in 5% bovine serum albumin (Sigma) in tris-buffered-saline (TBS) containing 0.01% Tween-20 (Sigma) for 1 h at room temperature. Incubation with anti-human CD63 (ab68418, Abcam, United Kingdom, 1/1000 in TBS-T) was carried out overnight at 4°C, thereafter the blots were washed three times for 10 min in TBS-T and incubated thereafter in secondary antibody (HRP-conjugated anti rabbit IgG1, 1/4000, BioRad) for 1 h at room temperature. The blots were washed five times for 10 min in TBS-T, followed by one wash in TBS before visualization with ECL (Amersham, United Kingdom). Membranes were imaged using the UVP transilluminator (UVP BioDoc-ITTM System, United Kingdom). For quantitative comparison of CD63 positive vesicles released from each cell line in the presence of CBD versus DMSO control, the amount of β-actin (ab20272, Abcam, 1/5000 in TBS-T) expression in the corresponding cell preparations was compared by densitometry, using ImageJ. The absence of actin in exosome samples was also tested to verify a lack of contamination by cellular debris in the exosome isolates.

### Western Blotting Analysis for Cellular Changes in Mitochondrial Associated Prohibitin and STAT-3 Expression in Response to CBD Treatment

Protein isolates from HEPG2, PC3 and MDA-MB231 cells were prepared, separated by SDS–PAGE and immunoblotted as described above (2.6). To assess changes in two mitochondrial associated proteins, prohibitin and STAT3, following CBD treatment, the membranes were incubated with anti-prohibitin antibody (ab75771, Abcam; 1/2000 in TBS-T) and anti-STAT3 (phospho Y705) antibody (ab76315, Abcam; 1/2000 in TBS-T). The secondary antibody was HRP-conjugated anti rabbit IgG1 (BioRad; 1/4000). For internal loading control, β-actin (ab20272, Abcam, 1/5000 in TBS-T) was used, and detection of prohibitin and STAT3 expression was normalized against β-actin expression by densitometry analysis using ImageJ.

### Cellular Respiration and Mitochondrial Function Analysis

Cellular respiration was measured in MDA-MB-231 and PC3 cancer cells using the Seahorse Bioanalyser according to the manufacturer’s instructions (Seahorse Biosciences, United States). The sensor cartridge was hydrated with Seahorse sensor media (Seahorse Biosciences) 18 h prior to the assay. In brief, mitochondrial respiration, as determined by oxygen consumption rate (OCR) was measured by seeding cells 2.5 × 10^4^ cells/well (for MDA-MB-231) or 4 × 10^4^ cells/well (for PC3) in specific 24 well Seahorse Bioanalyser plates (Seahorse Biosciences), 24 h prior to the cell respiration assay. Cells were treated with CBD (1 or 5 μM) for 1 h, followed by washing in Seahorse Assay medium (Seahorse Biosciences) supplemented with glucose and 1% sodium pyruvate, pH 7.4 at 37°C. Thereafter, oligomycin, carbonyl cyanide-4-(trifluoromethoxy) phenylhydrazone (FCCP, 0.2 μM) and antimycin/rotenone (0.25 μM) were added to the sensor plate prior to the commencement of calibration and the assay. Calculations were normalized to protein level, as calculated by Bradford assay directly after the experimental procedure. Each experiment was repeated 3–5 times, with technical replicates of four per plate.

### Effect of CBD on Cisplatin-Mediated Apoptosis of HEPG2 and MDA-MB231 Cancer Cells

HEPG2 and MDA-MB231 cells were grown as a monolayer in T75 flasks (Nunc, United States) until 80% confluent. The media was removed, the cells washed in DPBS and fresh medium added, containing 1 or 5 μM CBD, for 24 h. Medium containing 0.001% DMSO was used as control treatment. After 1 h incubation with the compounds, the media was removed, cells gently washed with DPBS and incubated with 100 μM cisplatin (Sigma, United Kingdom), dissolved in culture media, for further 24 h. Cell viability assessment was carried out by MTT assay. The optical density was measured as a percentage of untreated cells and repeated 3–5 times per cell type for experimental replicates, with five technical replicates per plate.

### Statistical Analysis

Graphs were prepared and statistical analysis performed using GraphPad Prism version 6 (GraphPad Software, San Diego, CA, United States). A one-way ANOVA was performed with Tukey’s *post hoc* analysis. Differences were considered significant for *p* ≤ 0.05 (^∗^*p* ≤ 0.05; ^∗∗^*p* ≤ 0.01; ^∗∗∗^*p* ≤ 0.001; ^∗∗∗∗^*p* ≤ 0.0001).

## Results

### Effects of CBD on Cancer Cell Viability

Cancer cell viability was not significantly affected by the levels of CBD used in these experiments after 1 h incubation (**Figure 1**). In PC3 cells, 1 μM CBD resulted in a 5.6% decrease in cell viability (*p* = 0.1583), and 5 μM CBD in a 2.2% decrease in cell viability (*p* = 0.7247) compared to DMSO treated control cells. The same was observed for HEPG2 cells, with both 1 and 5 μM CBD causing 1.18% decreased cell viability (*p* = 0.1890 and *p* = 0.2746, respectively) compared to DMSO treated control cells. CBD did also not affect MDA-MB-231 cell viability significantly compared to DMSO treated control cells, with a 3.5% decrease observed in 1 μM CBD (*p* = 0.7090) and 5.4% decrease in 5 μM CBD treated cells (*p* = 0.3081). In comparison, cell viability was affected to some extent by Cl-amidine (50 μM), which so far has proven our most effective EMV inhibitor with the lowest toxicity levels compared to other inhibitors previously tested (Kosgodage et al., 2017). Cell viability for PC3 cells was reduced by 20% (*p* = 0.0005), by 11% for HEPG2 (*p* = 0.0033) and by 5.3% for MDA-MB-231 (*p* = 0.0353) in the presence of 50 μM Cl-amidine compared to PBS treated control cells (**Figure 1**). In addition, longer-term (24 h) treatment effects of CBD on cancer cell viability was further assessed for HEPG-2 and MDA-MB-231 cells, showing dose-depended reduction in cell viability compared to control DMSO treated cells as follows: In HEPG2 cells, 1 μM CBD resulted in a 38.8% decrease in cell viability (*p* < 0.001), and 5 μM CBD in a 47.2% decrease in cell viability (*p* < 0.001) compared to DMSO treated control cells. In MDA-MB-231 cells, 1 μM CBD resulted in a 12.9% decrease in cell viability (*p* < 0.05), and 5 μM CBD in a 35.8% decrease in cell viability (*p* < 0.01) compared to DMSO treated control cells (**Supplementary Figure 2**).

**FIGURE 2:**
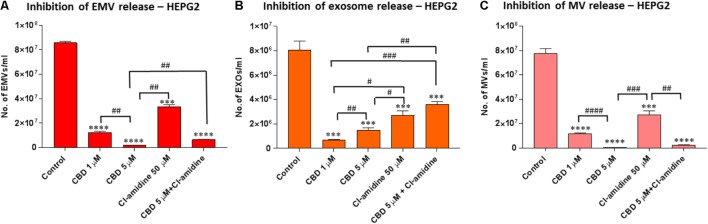
CBD significantly inhibits total EMV, exosome and MV release from HEPG2 cells. Inhibitory effects of CBD alone and in combination with Cl-amidine on extracellular vesicle release from HEPG2 cancer cells are presented as histograms which are based on size exclusion analysis by Nanosight Tracking Analysis (NTA). EMVs represent all vesicles 0–900 nm **(A)**; exosomes are vesicles <100 nm **(B)**; and microvesicles (MV) are 100–900 nm **(C)**. The experiments were repeated three times and the data presented are mean ± SEM of the results (^∗^*p* ≤ 0.05; ^∗∗^*p* ≤ 0.01; ^∗∗∗^*p* ≤ 0.001; ^∗∗∗∗^*p* ≤ 0.0001 versus Control; Differences between CBD and Cl-amidine treatment group is further indicated as #*p* ≤ 0.05; ##*p* ≤ 0.01; ###*p* ≤ 0.001; ####*p* ≤ 0.0001).

### EMV Release Profiles Vary Between PC3, HEPG2, and MDA-MB-231 Untreated Cancer Cells

A range in the total amount of EMVs (<900 nm) released from the three cancer cell lines used in this study varied considerably under normal control conditions (untreated cells in the absence of CBD, Cl-amidine or DMSO; **Supplementary Figure 3**). Differences were observed in the proportions of exosomes (<100 nm) and microvesicles (MVs; 100–900 nm) released from control treated cells (absence of EMV inhibitors CBD and/or Cl-amidine). While PC3 cells released the highest amount of EMVs and similar proportions of exosomes and MVs, both HEPG2 and MDA-MB-231 released a higher proportion of MVs versus exosomes (**Supplementary Figures 3A,B**). In addition, a range in the proportional release of the two MV subsets at 100–200 nm and 201–500 nm were observed between the three cell lines, particularly regarding the 201–500 nm subset which was proportionally highest in HEPG2 compared to PC3 and MDA-MB-231 cells (**Supplementary Figure 3B**).

**FIGURE 3:**
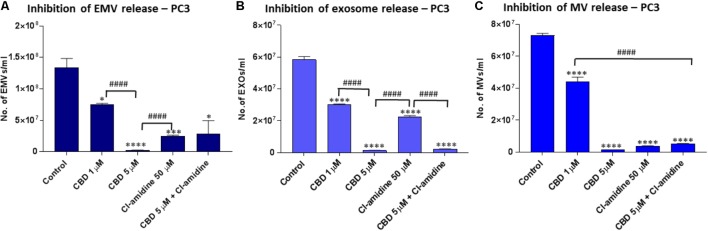
CBD significantly inhibits total EMV, exosome and MV release from PC3 cells. Inhibitory effects of CBD alone and in combination with Cl-amidine on extracellular vesicle release from PC3 cancer cells are presented as histograms which are based on size exclusion analysis by Nanosight Tracking Analysis (NTA). EMVs represent all vesicles 0–900 nm **(A)**; exosomes are vesicles <100 nm **(B)**; and microvesicles (MV) are 100–900 nm **(C)**. The experiments were repeated three times and the data presented are mean ± SEM of the results (^∗^*p* ≤ 0.05; ^∗∗^*p* ≤ 0.01; ^∗∗∗^*p* ≤ 0.001; ^∗∗∗∗^*p* ≤ 0.0001 versus Control; Differences between CBD and Cl-amidine treatment group is further indicated as #*p* ≤ 0.05; ##*p* ≤ 0.01; ###*p* ≤ 0.001; ####*p* ≤ 0.0001).

### CBD Effectively Inhibits Exosome and Microvesicle Release From HEPG2 Cells

Pre-treatment of HEPG2 with both 1 and 5 μM CBD, for 60 min before EMV isolation, resulted in a significant reduction of total EMV release compared to the DMSO treated control cells (86.7%; *p* = 0.0001 and 97.9%; *p* = 0.0002, respectively) and was more potent than for Cl-amidine (61.9%; *p* = 0.0002) compared to control. When using CBD (5 μM) in combination with Cl-amidine, a significantly higher inhibition was observed compared to Cl-amidine alone (*p* = 0.0058). Compared to control treated cells the combinatory treatment resulted in a 91.9% reduction of EMVs (*p* = 0.0002; **Figure 2A**).

Further analysis of the NTA data, based on size exclusion, was performed to elucidate the inhibitory effects of CBD on the release of exosome-sized vesicles (<100 nm) or MV-sized vesicles (≥100 nm) (**Figures 2B,C**). The total EMV vesicles collected at 25,000 *g* had been confirmed to be comprised of MVs and exosomes, as confirmed by separate isolation of MVs (centrifugation at 11,000 *g*) and of exosomes (100,000 *g*) as identified by the expression of CD63 (strong in exosomes, negligible in MVs), by phosphatidylserine exposition (higher in MVs compared to exosomes), and by electron microscopy (MVs ≥ 100 nm; exosomes <100 nm) according to previously established protocols [11,14,15, 81,82; see **Supplementary Figure 1**].

Analysis of inhibitory effects on exosome sized vesicles (<100 nm) showed that both concentrations of CBD (1 and 5 μM) were more effective (91.6%; *p* = 0.0005 and 84.0%; *p* = 0.0009; respectively) than Cl-amidine (68.4%; *p* = 0.0026). The lower dose of CBD (1 μM) was the most potent inhibitor of exosome release in this cancer cell type. Combinatory treatment with 5 μM CBD and Cl-amidine resulted in less exosome inhibition (57.9% compared to control; *p* = 0.0039) than any of the single inhibitor treatments, albeit not statistically significantly different from Cl-amidine treatment alone (*p* = 0.1134), while significantly less compared to CBD alone (*p* = 0.0039 for 1 μM CBD; *p* = 0.0025 for 5 μM CBD; **Figure 2B**).

The inhibitory effect of CBD on MV-sized vesicle release (≥100 nm) was significant in HEPG2 cells for both 1 μM (86.1%; *p* = 0.0001) and 5 μM (99.6%; *p* = 0.0001) concentrations of CBD compared to control cells, with 5 μM CBD being significantly more effective (*p* = 0.0001). MV inhibitory effects of Cl-amidine in comparison were 61.1% compared to control (*p* = 0.0007), while combinatory treatment of CBD (5 μM) and Cl-amidine showed a similar effect on total MV release (96.2%; *p* = 0.0001) as CBD alone (**Figure 2C**).

The histograms from the NTA analysis showed a notable reduction in the approximately 300 nm (201–400 nm range) peak in CBD pre-treated HEPG2 cells (**Supplementary Figure 4**), a feature also observed in the combinatory treatment with CBD and Cl-amidine, while this 300 nm (201–400 nm range) peak was present in Cl-amidine treated cells (**Supplementary Figure 4**). Thus the effect of CBD and Cl-amidine on MV release in the 100–200 and 201–500 nm ranges was further assessed in all three cell lines used in this study (**Figure 5**).

**FIGURE 4:**
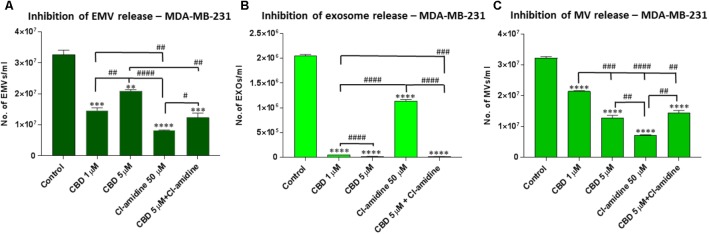
CBD significantly inhibits total EMV, exosome and MV release from MDA-MB-231 cells. Inhibitory effects of CBD alone and in combination with Cl-amidine on extracellular vesicle release from MDA-MB-231 cancer cells are presented as histograms which are based on size exclusion analysis by Nanosight Tracking Analysis (NTA). EMVs represent all vesicles 0–900 nm **(A)**; exosomes are vesicles <100 nm **(B)**; and microvesicles (MV) are 100–900 nm **(C)**. The experiments were repeated three times and the data presented are mean ± SEM of the results (^∗^*p* ≤ 0.05; ^∗∗^*p* ≤ 0.01; ^∗∗∗^*p* ≤ 0.001; ^∗∗∗∗^*p* ≤ 0.0001 versus Control; Differences between CBD and Cl-amidine treatment group is further indicated as #*p* ≤ 0.05; ##*p* ≤ 0.01; ###*p* ≤ 0.001; ####*p* ≤ 0.0001).

**FIGURE 5:**
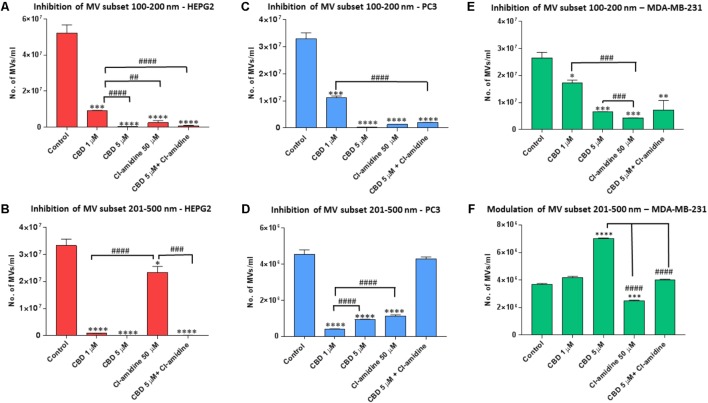
CBD modulates different MV subpopulations released from PC3, HEPG2 and MDA-MB-231 cells. Inhibitory effects of CBD alone, Cl-amidine or CBD in combination with Cl-amidine on 100–200 nm and 201–500 nm sized microvesicles, based on size exclusion analysis by Nanosight Tracking Analysis (NTA). Inhibition of 100–200 nm MV release in **(A)** PC3; **(C)** HEPG2; and **(E)** MDA-MB-231 cancer cells. Inhibition of 201–500 nm MV release in **(B)** PC3; **(D)** HEPG2; and **(F)** MDA-MB-231 cancer cells. The experiments were repeated three times and the data presented are mean ± SEM of the results (^∗^*p* ≤ 0.05; ^∗∗^*p* ≤ 0.01; ^∗∗∗^*p* ≤ 0.001; ^∗∗∗∗^*p* ≤ 0.0001 versus Control; Differences between CBD and Cl-amidine treatment group is further indicated as #*p* ≤ 0.05; ##*p* ≤ 0.01; ###*p* ≤ 0.001; ####*p* ≤ 0.0001).

While CBD had a strong inhibitory effect on both MV subsets, Cl-amidine had a much stronger inhibitory effect on the smaller (100–200 nm) than larger (201–500 nm) MV subset compared to control treated HEPG2 cells (**Figures 5A,B**). In the smaller MV subset, 5 μM CBD showed a stronger inhibitory effect (99.4%; *p* = 0.0003) than 1 μM CBD (82.0%; *p* = 0.0006) compared to control. Cl-amidine reduced this smaller subset by 98.1% (*p* = 0.0004) compared to control, and combinatory treatment of 5 μM CBD and Cl-amidine had a 98.2% inhibitory effect (*p* = 0.0003) compared to control treated cells (**Figure 5A**).

For the shedding of the larger 201–500 nm sized vesicles, CBD was a more effective inhibitor in HEPG2 cells than Cl-amidine, with 1 μM CBD showing 97.1% (*p* = 0.0001) and 5 μM CBD 100% (*p* = 0.0002) inhibitory effect, respectively, compared to controls. In this larger MV subset, Cl-amidine showed only 30% inhibition (*p* = 0.0356) compared to control. The combinatory application of 5 μM CBD and Cl-amidine had a similar inhibitory effect (99.7%; *p* = 0.0001) as 5 μM CBD alone (**Figure 5B**).

### CBD Effectively Inhibits Exosome and Microvesicle Release From PC3 Cells

Pre-treatment of PC3 with both 1 and 5 μM CBD, for 60 min before EMV isolation, resulted in a significant reduction of total EMV release compared to the DMSO treated control cells (44.5 and 98.1% reduction of EMV release for 1 and 5 μM CBD, respectively; *p* = 0.0149; *p* = 0.0008, respectively) (**Figure 3A**). The inhibitory effect by 5 μM CBD on total EMV release was greater than observed with our previously most efficient EMV inhibitor Cl-amidine, which was used for comparison (*p* = 0.0001), while Cl-amidine had a significantly stronger EMV inhibitory effect than 1 μM CBD (*p* = 0.0001). When using CBD (5 μM) in combination with Cl-amidine no additive change in total EMV inhibition was found compared to single inhibitors (**Figure 3A**).

Analysis of inhibitory effects on exosome sized vesicles (<100 nm), showed that both CBD and Cl-amidine significantly reduced the number of vesicles released compared to control, untreated PC3 cells (98.0 versus 66.1%; *p* = 0.0001 and *p* = 0.0001, respectively compared to control). A significantly stronger inhibitory effect was observed for 5 μM CBD than with Cl-amidine (*p* = 0.0001), while 1 μM CBD was less effective, inhibiting exosome release by 51.3% compared to control (*p* = 0.0002). Combinatory treatment with 5 μM CBD and Cl-amidine gave similar results as single CBD (5 μM) inhibitor treatment (96.6%; *p* = 0.0001 compared to control) (**Figure 3B**).

The inhibitory effect of CBD on MV-sized vesicle release (≥100 nm) was significant for both 1 μM (38.5%; *p* = 0.0009) and 5 μM (98.1%; *p* = 0.0001) concentrations of CBD, compared to non-treated control cells, although 5 μM CBD was significantly more effective than 1 μM CBD (*p* = 0.0002). The effect of 5 μM CBD alone was similar in reducing in MV release as seen for Cl-amidine compared to control cells (95.6%; *p* = 0.0001) while combinatory treatment of CBD (5 μM) and Cl-amidine did not show a further significant additive effect on MV release (93.6%; *p* = 0.0001 compared to control) (**Figure 3C**).

Next, the effect of CBD and Cl-amidine on MV release in the 100–200 and 201–500 nm ranges was further assessed for PC3 cells (**Figures 5C,D**). MV count in the size range of 100–200 nm was significantly reduced at similar levels by 5 μM CBD, Cl-amidine, and CBD (5 μM) in combination with Cl-amidine, compared to DMSO treated controls (98.8, 95.9, and 94.4%, respectively; *p* = 0.0001 for all groups compared to control). CBD at 1 μM also showed significant inhibition compared to control (71.8%; *p* = 0.0007), but significantly less inhibition on this MV subset than 5 μM CBD alone, Cl-amidine alone or CBD (5 μM) and Cl-amidine in combination (*p* = 0.0001, *p* = 0.0001 and *p* = 0.0002, respectively; **Figure 5C**).

For the shedding of the larger MV subset of 201–500 nm sized vesicles, CBD was more effective at the lower dose of 1 μM than at 5 μM (*p* = 0.0001). Cellular release of this MV subset was reduced by 92% in 1 μM CBD treated cells (*p* = 0.0001), and by 81.2% in 5 μM CBD treated cells (*p* = 0.0001) compared to controls, while Cl-amidine alone reduced this subset of MVs by 64.0% (*p* = 0.0002). When used in combination, 5 μM CBD with Cl-amidine did not show significant inhibition of this MV subset compared to control (4% inhibition; *p* = 0.4250) (**Figure 5D**).

### CBD Effectively Inhibits Exosome and Microvesicle Release From MDA-MB-231 Cells

Pre-treatment of MDA-MB-231 with both 1 and 5 μM CBD for 60 min before EMV isolation resulted in a significant reduction of total EMV release compared to the control treated cells (53.4%; *p* = 0.0001 and 42.9%; *p* = 0.0001, respectively) but was a less potent total EMV inhibitor than Cl-amidine (75.9%; *p* = 0.0001 compared to control). When using CBD (5 μM) in combination with Cl-amidine, a significantly (*p* = 0.0052) higher inhibition was observed compared to 5 μM CBD alone, while there was no significant difference compared to 1 μM CBD treatment (*p* = 0.2474). Compared to control treated cells the combinatory treatment resulted in a 55.1% reduction of total EMVs (*p* = 0.0006) (**Figure 4A**)

Analysis of inhibitory effects on exosome sized vesicles (<100 nm) showed that both concentrations of CBD (1 and 5 μM) were similarly potent at being more effective inhibitors (97.5%; *p* = 0.0001 and 99%; *p* = 0.0001; respectively) than Cl-amidine (46.7%; *p* = 0.0001) compared to control treated MDA-MB-231 cells. Combinatory treatment with 5 μM CBD and Cl-amidine resulted in similar effects on exosome inhibition as CBD alone (99.5%; *p* = 0.0001) (**Figure 4B**).

The inhibitory effect of CBD on MV-sized vesicle release (≥100 nm) was significant for both concentrations, albeit less effective than Cl-amidine. CBD showed 34.4% (*p* = 0.0001) inhibition at 1 μM, and 56.5% (*p* = 0.0001) inhibition at 5 μM, compared to control, with 5 μM CBD being a significantly more effective (*p* = 0.0007) total MV inhibitor. In comparison, MV inhibitory effects of Cl-amidine were higher, at 77.8% compared to control (*p* = 0.0001), while combinatory treatment of CBD (5 μM) and Cl-amidine showed a similar effect on total MV release (52.7%; *p* = 0.0001) as 5 μM CBD alone (**Figure 4C**)

The effect of CBD and Cl-amidine on MV release in the 100–200 and 201–500 nm ranges was further assessed in MDA-MB-231 cells (**Figures 5E,F**). While both concentrations of CBD showed a significant decrease on the smaller (100–200 nm) MV subset, the 5 μM CBD concentration showed a stronger inhibitory effect (77.0%; *p* = 0.0007) than 1 μM CBD (41.7%; *p* = 0.0174), compared to control. Cl-amidine had the strongest inhibitory effect on this MV subset (84.8%; *p* = 0.0004), while the combination of CBD and Cl-amidine showed no significant change (*p* = 0.4238) compared to 5 μM CBD alone, reducing this MV subset by 61.0% compared to control (*p* = 0.0089) (**Figure 5E**).

For the shedding of the larger 201–500 nm sized vesicles, less inhibitory effects were observed for CBD. Neither CBD alone nor in combination with Cl-amidine, showed any inhibitory effects, while Cl-amidine alone reduced the release of this MV population by 25.7% (*p* = 0.0004). Contrary to what was observed in the other two cancer cell lines, CBD increased the release of this MV subpopulation by 10.5% (*p* = 0.0143) at 1 μM concentration and by 84.2% (*p* = 0.0001) at 5 μM concentration compared to control – an effect that was somewhat counteracted in the combinatory treatment with Cl-amidine, where this CBD-mediated increase was reduced by 42.3% (*p* = 0.0001), bringing it down to similar levels as the control treated cells (**Figure 5F**).

### CBD Modulates CD63 Expression in HEPG2, PC3, and MDA-MB-231 Cells

**FIGURE 6:**
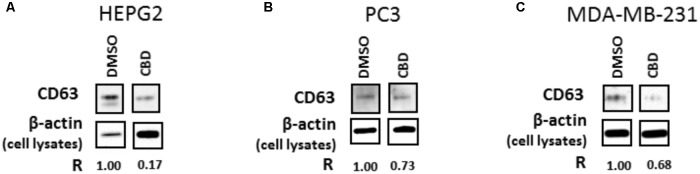
CD63 exosomal marker is reduced following 1 h CBD treatment in HEPG2, PC3, and MDA-MB-231 cancer cells. The results from the NTA analysis were confirmed by Western blotting for the exosomal CD63 marker, which was reduced in all three cell lines following 1 h treatment with 5 μM CBD: **(A)** CD63 expression is reduced in CBD treated versus DMSO control treated HEPG2 cancer cells; **(B)** CD63 expression is reduced in CBD treated versus DMSO control treated PC3 cells; **(C)** CD63 expression is reduced in CBD treated versus DMSO control treated MDA-MB-231 cells. All EMV preparations were performed in equal buffer volume (50 μl) and all cell lysates were prepared in equal buffer volume (50 μl) between all samples, for accurate presentation of amounts of vesicles isolated and amounts of cells grown and collected per flask. For EMV isolates, 20 μl of sample was loaded per lane, while for β-actin detection in the corresponding cell lysates, 10 μl of sample was loaded per lane. The relative detection of CD63 in EMVs released from the corresponding cell preparation is indicated by “R,” in relation to β-actin detection in the corresponding cell isolate, for comparison between CBD treatments versus DMSO control.

Findings from the NTA analysis, showing significant reduction in EMV release, particularly exosome release, was further assessed by Western blotting of CD63 expression in all three cancer cell lines following CBD treatment (5 μM). The expression of CD63 was reduced in all three cell lines following 1 h CBD treatment (**Figure 6**), thus confirming the NTA results, showing significant reduction in exosome biogenesis in response to CBD treatment in HEPG2 (**Figure 6A**), PC3 (**Figure 6B**) and MDA-MB-231 (**Figure 6C**) cancer cells. The absence of actin in exosome samples was also confirmed to exclude contamination by cellular debris in the exosome isolates (not shown).

### Mitochondrial Function Alteration Analysis in MDA-MB-231 and PC3 Cells Following CBD Treatment

**FIGURE 7:**
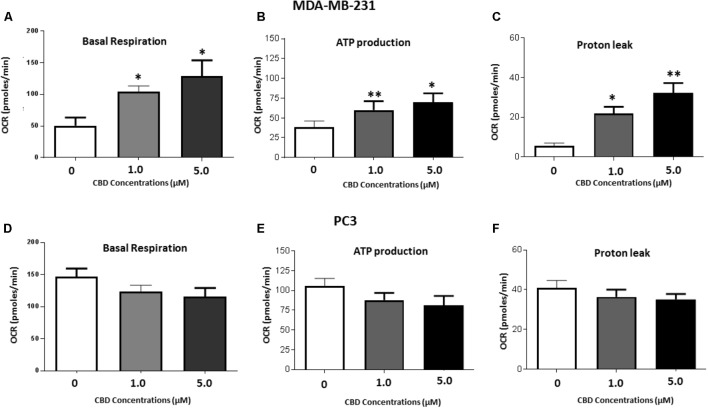
Mitochondrial function alteration following 1 h CBD treatment in MDA-MB-231 and PC3 cancer cells. MDA-MB-231 and PC3 cells were treated with 1 or 5 μM CBD for 1 h prior to mitochondrial functional analysis using the Seahorse Bioanalyser for the following parameters: **(A,D)** Basal mitochondrial respiration; **(B,E)** Quantification of ATP production; **(C,F)** Proton leak. Data shown is repeated three times (for MDA-MB-231 cells) or five times (for PC3 cells) with four technical replicates per plate. Data is represented as mean ± SEM. ^∗^*p* < 0.05, ^∗∗^*p* < 0.01, ^∗∗∗^*p* < 0.001 versus untreated control cells.

Mitochondrial analysis, using the Seahorse Bionalayser, measured mitochondrial respiration along with several key mitochondrial factors associated with mitochondrial function through oxygen consumption (**Figure 7**). In MDA-MB-231 cells, basal mitochondrial OCR (oxygen consumption rate) was significantly increased, compared to non-treated controls (50.4 ± 13.5 pMoles/min), following 1 h CBD treatment at 1 μM (104.1 ± 23.7 pMoles/min; *p* ≤ 0.05) and 5 μM CBD (129.6 ± 36.4 pMoles/min; *p* ≤ 0.05) (**Figure 7A**). In PC3 cells, basal mitochondrial OCR showed a decreasing trend with increased dose of CBD, following 1 h CBD treatment at 1 μM (124.2 ± 9.6 pMoles/min; *p* ≤ 0.05) and 5 μM CBD (116.3 ± 13.1 pMoles/min), while not statistically significant compared to non-treated control (146.8 ± 12.9 pMoles/min) (**Figure 7D**).

Dose dependent changes in relative ATP production levels were observed in both cancer cell lines in response to CBD treatment compared to untreated cells. Compared to untreated MDA-MB-231 cells (37.5 ± 8.9 pMoles/min), in 1 μM CBD treated MDA-MB-231 cells ATP production levels were 60.5 ± 12.8 pMoles/min (*p* ≤ 0.05) and in 5 μM CBD treated cells ATP production levels were 69.2 ± 16.8 pMoles/min (*p* ≤ 0.05) (**Figure 7B**). PC3 cells showed a decreasing trend in ATP with increased dose of CBD, albeit not statistically significant compared to control treated PC3 cells (106.0 ± 9.4 pMoles/min). In 1 μM CBD treated PC3 cells ATP production levels were 87.8 ± 9.1 pMoles/min and in 5 μM CBD treated cells ATP production levels were 81.2 ± 11.9 pMoles/min (**Figure 7E**).

A significant dose dependent increase in proton leak was observed for both concentrations of CBD in MDA-MB-231 cells as follows: 1 μM CBD: 21.6 ± 3.2 pMoles/min (*p* ≤ 0.01), and 5 μM CBD: 32.2 ± 9.5 pMoles/min (*p* ≤ 0.01), compared to untreated cells (5.8 ± 3.3 pMoles/min) (**Figure 7C**). In PC3 cells proton leak was somewhat, but not significantly, reduced in the presence of 1 μM (36.4 ± 3.6 pMoles/min) and 5 μM CBD (35.1 ± 2.8 pMoles/min) compared to untreated cells (40.9 ± 3.7 pMoles/min) (**Figure 7F**).

### CBD Modulates Expression of Mitochondrial Associated Proteins Prohibitin and STAT3

**FIGURE 8:**
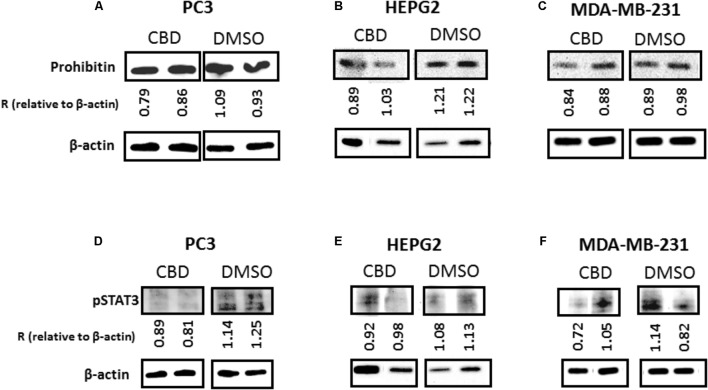
CBD modulates expression of prohibitin and STAT3 in cancer cells. PC3, HEPG2, and MDA-MB-231 cells were tested for changes in mitochondrial associated prohibitin and STAT3 expression following 1 h treatment with CBD (5 μM). Levels of prohibitin were reduced in all three cancer cell lines while this reduction was more marked in PC3 **(A)** and HEPG2 **(B)** cells compared to MDA-MB-231 **(C)**. STAT3 (phospho Y705) was also reduced after 1 h CBD (5 μM) in both PC3 **(D)** and HEPG2 cells **(E)**, while MDA-MB-231 cells showed a similar, albeit less marked trend **(F)**. Beta-actin is shown as an internal loading control and “R” indicates the change of prohibitin and pSTAT3 expression relative to β-actin levels, respectively, for comparison between CBD treatment and DMSO control.

Protein isolates from HEPG2, PC3 and MDA-MB231 cells were further assessed for changes in two mitochondrial associated proteins; prohibitin and STAT3, following CBD (5 μM) treatment and compared to DMSO treated controls. In all three cancer cell lines, levels of prohibitin were reduced, although more marked changes were noted in the PC3 (**Figure 8A**) and HEPG2 (**Figure 8B**) cells compared to the MDA-MB-231 cells (**Figure 8C**). In all three cancer cell lines, STAT3 (phospho Y705) was also reduced after 1 h CBD (5 μM) treatment; again this reduction was higher in PC3 and HEPG2 cells (**Figures 8D,E**), compared to the MDA-MB-231 cells (**Figure 8F**).

### CBD Sensitizes HEPG2 and MDA-MB-231 Cancer Cells to on Cisplatin-Mediated Apoptosis

**FIGURE 9:**
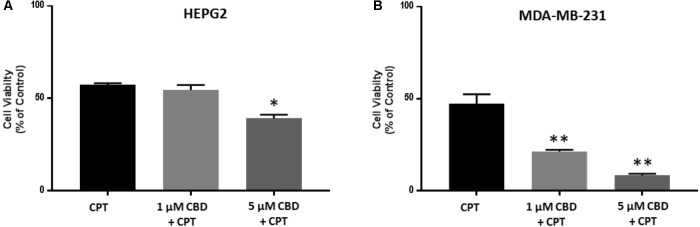
CBD sensitizes HEPG2 and MD-MB-231 cancer cells to cisplatin-mediated apoptosis. HEPG2 **(A)** and MDA-MB-231 **(B)** cells were treated with 1 or 5 μM CBD for 24 h prior to further 24 h incubation with cisplatin (CSP, 100 μM). Cell viability was assessed by MTT assay. Data shown is repeated three times with three technical replicates per plate. Data is represented as mean ± SEM. ^∗^*p* < 0.05, ^∗∗^*p* < 0.01 versus untreated control cells.

In both HEPG2 and MDA-MB-231 cancer cells, CBD increased cisplatin-mediated apoptosis (**Figure 9**). In HEPG2 cells, compared to untreated control cells, cisplatin treatment alone resulted in 57.3% cell viability (*p* < 0.01). However, this effect was significantly enhanced (*p* < 0.001) if cells were first treated with 1 and 5 μM CBD (54.5 and 39.1%, respectively), prior to cisplatin (**Figure 9A**). In MDA-MB-231 cells, compared to untreated control cells, cisplatin treatment alone resulted in 47.3% cell viability (*p* < 0.001), while 21.3 and 8.3% cell viability was observed for cells treated with 1 or 5 μM CBD prior to cisplatin treatment (*p* < 0.01). CBD treatment alone led to significant changes in cell viability, but to a much lesser extent than those observed when cells were first treated with CBD followed by cisplatin (**Figures 9A,B**).

## Discussion

This study reveals a novel finding for CBD; it can selectively inhibit the release of subsets of EMVs, from cancer cell lines. The different cancer cell lines tested here (prostate cancer PC3, hepatocellular carcinoma HEPG2 and breast adenocarcinoma MDA-MB-231) varied in the proportional amounts of total EMVs, MVs and exosomes released under standard conditions (**Supplementary Figure 3**). Nonetheless, across this range of EMV release profiles, we found that CBD consistently inhibited exosome release significantly and also had significant, albeit more variable, modulating effects on MV release. This novel function of CBD on EMV release, revealed here for the first time, may be of high relevance for optimized therapeutic application in various EMV-mediated pathologies.

There is a considerable interest in using EMV inhibitors to sensitize cancers to chemotherapy. Previous work, using the calpain inhibitor calpeptin for MV inhibition, in combination with chemotherapy drugs fluorouracil and docetaxel, reduced the effective chemotherapeutic dose needed by 100-fold to produce comparable reduction in tumor volume *in vivo*. The same study also showed that methotrexate is released from cancer cells in MVs (Jorfi et al., 2015). Similar findings of drug efflux and sensitisation to gemcitabine in response to MV inhibition were established in pancreatic cancer *in vitro* and *in vivo* upon MV inhibition via ERK-mediated pathways (Muralidharan-Chari et al., 2016); and to doxorubicin and pixantrone treatment upon exosome inhibition via inhibition of ATP-transporter A3 in B-cell lymphoma models (Koch et al., 2016). Also, chemotaxis of cancer cells has been shown to be promoted by exosome secretion, but to be diminished by knockdown of the exosome regulator Rab27a (Sung and Weaver, 2017). Inhibition of exosome secretion has been shown to cause defective tumor cell migration (Sung et al., 2015), while exosomes isolated from gastric tumor cells were shown to induce tumor cell migration and promotion in receiving cells (Wu et al., 2016). Previously, our work identified a novel pathway of MV release involving peptidylarginine deiminases (PADs) and the effective inhibition of PAD-mediated EMV release using Cl-amidine (Kholia et al., 2015; Kosgodage et al., 2017), suggesting implications in a number of pathologies (Lange et al., 2017). In addition, we have also recently shown that several new candidate EMV inhibitors, including bisindolylmaleimide-I, imipramine and Cl-amidine, are more potent EMV inhibitors than calpeptin (Kosgodage et al., 2017) and those they sensitize cancer cells to chemotherapeutic agents. This further call for the identification of novel EMV inhibitors, which are safe for application *in vivo*, such as CBD now identified here. Indeed, as we have shown here, by significantly increasing cisplatin mediated apoptosis, CBD showed a similar ability to other EMV inhibitors of sensitizing cancer cells to chemotherapy.

CBD-mediated inhibition of EMV release, observed in the present study, was more effective for some EMV subsets and cancer cells than Cl-amidine, our most potent EMV inhibitor to date (Kosgodage et al., 2017). One intriguing finding in our study is the selectivity of CBD on different EMV subsets in the three different cancer cell lines, which also varied with concentration (1 and 5 μM). In PC3 cells, 5 μM of CBD was the most effective inhibitor of total EMVs, exosomes, total MVs and the smaller MV subset (100–200 nm), while 1 μM CBD was most effective at inhibiting the larger MV subset (201–500 nm). In the HEPG2 hepatocellular carcinoma cells 5 μM CBD had the main impact on total EMV and MV release, while 1 μM CBD most significantly affected exosome release. Overall, the potency of CBD to inhibit all subsets of EMVs tested here was most marked in the HEPG2 cells. In MDA-MB-231 cells the inhibitory effect of CBD was particularly marked for exosome release, while total MV release was less inhibited by CBD compared to Cl-amidine. Recent studies in this invasive breast cancer cell line have suggested an active role for exosomes in increased cell movement and metastasis (Harris et al., 2015). The increase in MVs released in the size range of 201–500 nm in response to CBD treatment was specific for the MDA-MB-231 cells. This may indicate a higher sensitivity of this particular cancer cell line to CBD and may also be a sign of pseudoapoptotic responses, where increased membrane permeability and leakage of reactive oxygen species (ROS) and other apoptotic factors is still low enough for the cell to turn the apoptosome into MVs for export of hazardous agents (Mackenzie et al., 2005; Inal et al., 2013). Indeed, a dose-dependent increase in ROS levels in response to 1 h CBD treatment (**Supplementary Figure 5**) alongside a dose-dependent increase in proton leak, mitochondrial respiration and ATP levels (**Figures 7A–C**) were observed in this cancer cell line in particular. In the PC3 cells on the other hand, the reduced EMV release observed in all EMV subsets, tallied in with a trend of reduced ATP production and reduced proton leak as well as lowered mitochondrial respiration, indicating an absence of pseudoapoptotic responses, as clearly reflected also in the significant reduction of the 201–500 nm MV subset in the PC cancer cells.

In the current study we have found that while reducing EMVs, CBD also modulates mitochondrial function and the expression of mitochondrial associated proteins prohibitin and STAT3. Although studies on direct links between EMV release and mitochondrial changes are relatively limited, requiring further investigation, EMV generation has previously been linked to this organelle (Mackenzie et al., 2005; Qu et al., 2007; Lopez et al., 2008; Morel et al., 2010; Dubyak, 2012; Soto-Heredero et al., 2017). Both changes in mitochondrial calcium buffering and dynamics, including ROS, ATP and proton leak, have previously been shown to be linked to MV formation (Mackenzie et al., 2005) and to affect ATP-mediated release of MVs and exosomes (Qu et al., 2007; Dubyak, 2012). Mitochondrial stress can also lead to MV formation via pro-apoptotic Bax and Bak, which insert into the mitochondrial outer membrane resulting in its depolarisation and increased membrane permeability. This leads to ROS, cytochrome C (Cyt C) and apoptosis inducing factor (AIF) leakage into the cytoplasm and eventual apoptosis. Where apoptosis is triggered by the extrinsic pathway, such as stimulation of FasL, activation of caspase 8 leads to cleavage of Bid, tBid then translocating to the mitochondrial membrane, mediating Cyt C release; this causes cytoskeletal degradation and formation of the apoptosome (Dale and Friese, 2006; Lopez et al., 2008; Inal et al., 2012). In scenarios of minimal damage the cell can use the apoptosome to form a MV and export the hazardous agents via pseudoapoptosis (Inal et al., 2012). Furthermore, pseudoapoptosis has been shown to involve rapid reversible mitochondrial depolarization, mitochondrial swelling and changes in mitochondrial and cytosolic calcium (Mackenzie et al., 2005). In cancer cells, a previous study has for example shown a ten-fold increase in the release of a 333–385 nm MV subset in pseudoapoptotic response to sublytic C5b-9 stimulation (Stratton et al., 2015a). Mitochondrial permeability has been shown to be important also for MV shedding from platelets, where the natural phenol and Bax activator gossypol promoted mitochondrial depolarization, PS exposure and MV release (Dale and Friese, 2006). As it is now thought that many oncogenes and tumor suppressors control calcium flow into the mitochondrion, one key emerging target in cancer treatment is mitochondrial control of calcium signaling (Danese et al., 2017). Previously, effects of CBD on modulating mitochondrial calcium buffering and mitochondrial function have been described (Ryan et al., 2009; Mato et al., 2010; De Petrocellis et al., 2011; Shrivastava et al., 2011; Rimmerman et al., 2013; Fisar et al., 2014; Cui et al., 2017), including on mitochondrial swelling, ROS production and mitochondrial potential (Mato et al., 2010). STAT3 is indeed implicated in mitochondrial calcium control (Yang et al., 2015; Garama et al., 2016; Yang and Rincon, 2016) and the reduction in STAT3 in cancer cells observed here following CBD treatment may thus have modulatory effects on EMV release. A reduction of STAT3 by CBD has previously been shown in glioblastoma cells where it was for example related to the inhibition of self-renewal (Singer et al., 2015). Prohibitin is ubiquitously expressed in many cell types and involved amongst other in energy metabolism, proliferation and apoptosis (Peng et al., 2015; Ande et al., 2017). It acts as a scaffold protein in the inner mitochondrial membrane and is thus important for the regulation of mitochondrial architecture (Merkwirth et al., 2012). Prohibitin is critical for mitochondrial house-keeping including mitochondrial dynamics, morphology and biogenesis as well as stabilizing the mitochondrial genome (Peng et al., 2015). Here we show, for the first time, that prohibitin is reduced in cancer cells following CBD treatment. The slight variability in reduction of prohibitin in response to CBD between the cancer cell lines tested here tallies in with the observed differences in effectivity of CBD to inhibit EMVs from these different cancer cells. A similar correlation was found between expression changes in STAT3 and inhibition of EMV release following CBD treatment, as both PC3 and HEPG2 cells showed more reduction in STAT3 levels following CBD treatment, alongside a more pronounced inhibitory effect on total EMV release, compared to MDA-MB-231 cells; which, while showing overall significant reduction in EMVs and reduced levels of STAT3 and prohibitin, these effects were somewhat less marked than in the other two cancer types. The EMV modulatory effects of CBD could thus be partly mediated by the above observed mitochondrial changes. In addition, CBD has also been shown for example to stimulate mitochondrial uptake of calcium, followed by a decrease and a matching sudden increase in intracellular calcium (Ryan et al., 2009), indicating thus also putative dynamic effects on EMV release. Notably, in PC3 cells, a CBD-dose-dependent trend was observed for reduced ATP production, which correlated with the overall reduction observed in total EMVs, exosomes and MVs in response to CBD treatment, compared to DMSO treated control cells. Furthermore, prohibitin has previously been shown to protect cancer cells from ER stress and chemotherapy-induced cell death (Cheng et al., 2014; Tortelli et al., 2017). Prohibitin accumulation in mitochondria and *de novo* accumulation has been shown to cause chemoresistance, while knock-down of prohibitin sensitized cancer cells to chemotherapeutic treatment (Tortelli et al., 2017). Inhibition of prohibitin has also been shown to repress cancer cell malignancy progression in hypoxia (Cheng et al., 2014). The observed reduction in prohibitin observed here, following CBD treatment, may thus be an important factor in contributing to the sensitisation of cancer cells to chemotherapeutic agents, as previously shown for CBD in glioblastoma (Torres et al., 2011), in addition to affecting EMV release due to changes in mitochondrial function caused partly by prohibitin and STAT3 downregulation following CBD exposure.

Using a combined application of CBD (5 μM) with Cl-amidine resulted in different effects on the various EMV subsets and varied between the three cancer cell lines. In general, Cl-amidine did not have additive effects on the inhibitory effect of EMV release compared to CBD alone, while the combinatory treatment was more effective on some subsets than Cl-amidine alone, as was observed on exosome release in PC3 cells and on MV release in HEPG2 cells. However, the difference between cancer cell types to combinatory treatment did not significantly affect the larger MV subset in PC3 cells, while both CBD and Cl-amidine alone did. Similarly, combinatory treatment did not show more effect than CBD or Cl-amidine alone on exosome release from HEPG2 cells. Interestingly, in MDA-MB-231 cells, Cl-amidine counteracted the increased CBD-mediated release observed for the larger MV subset (201–500 nm), when used in combination, bringing the amount of vesicles release down to similar levels as for the control treated cells. Overall our results suggest that the two EMV inhibitors act on different pathways involved in MV and exosome release. While previously, Cl-amidine has been shown to act on MV biogenesis via increased cytoskeletal actin deimination and nuclear PAD translocation, indicative for changes in histone deimination (Kholia et al., 2015), CBD may act in part through modulation of mitochondrial metabolism as described here. Accordingly, and depending on which EMV subset is being targeted, our results indicate that tailored approaches for selective EMV inhibition could be developed for various EMV mediated pathologies. The expanded repertoire of EMV inhibiting agents, including CBD now revealed here, along with its sensitizing effects on cancer cells to cisplatin-mediated apoptosis, indicates a therapeutic potential for sensitisation of cancer cells to chemotherapy, as has been demonstrated for other promising EMV inhibitors (Tang et al., 2012; Federici et al., 2014; Jorfi et al., 2015; Koch et al., 2016; Muralidharan-Chari et al., 2016; Kosgodage et al., 2017). Importantly, such EMV-modulating agents could be used to allow for lower dose of chemotherapeutic drug for effective inhibition of tumor growth *in vivo* (Jorfi et al., 2015; Muralidharan-Chari et al., 2016). The ability of CBD to inhibit EMV release may indeed be a hitherto overlooked contributing factor in the beneficial effects of CBD observed in cancer therapy, where the exact mechanisms still remain to be unraveled (Torres et al., 2011; Ramer et al., 2012, 2014; Massi et al., 2013; Vara et al., 2013; Haustein et al., 2014; Velasco et al., 2016; Pisanti et al., 2017), as for example in glioma models, where CBD has been shown to enhance effects of temozolomide (Torres et al., 2011). Modulating EMV release may thus be an important therapeutic approach, also to prevent metastasis, where tumor derived exosomes have been shown to be involved in preparation of the pre-metastatic niche (Hoshino et al., 2015).

## Conclusion

A new mode of action for CBD in cancer, via modulation of EMV release, is revealed here for the first time. The findings presented in this study serve as a first proof of principle for CBD-mediated inhibition and modulation of EMV biogenesis, and shows cancer-type and dose specific effects. As CBD modulation of mitochondrial functions is well established, the effects observed here on changes in EMV release, mitochondrial function and mitochondrial associated proteins, alongside sensitisation of cancer cells to cisplatin mediated apoptosis, provide a platform for further research on detailed mechanistic pathways of CBD’s mode of action on EMV biogenesis and cellular communication. Furthermore, this work opens up wide ranging research into novel therapeutic avenues in EMV-mediated pathologies.

## Author Contributions

UK, RM, AH, and SL carried out the experiments. AN, GG, ET, JI, JB, and SL contributed to experimental design and data analysis. SL and AN wrote the manuscript. All authors contributed equally to the critical reviewing of the manuscript.

## Conflict of Interest Statement

GG is founder and chairman of GW Pharmaceuticals. AN is a scientific advisor to GW Pharmaceuticals. The remaining authors declare that the research was conducted in the absence of any commercial or financial relationships that could be construed as a potential conflict of interest.
