# Supplementary material for: Cannabidiol (CBD) Is a Novel Inhibitor for Exosome and Microvesicle (EMV) Release in Cancer
Source: Front Pharmacol. 2018 Aug 13;9:889. doi: 10.3389/fphar.2018.00889 (PMC6099119; doi:10.3389/fphar.2018.00889)

Supplementary Material

Cannabidiol (CBD) is a Novel Inhibitor for Exosome and Microvesicle (EMV) Release in Cancer

Uchini S. Kosgodage[1], Rhys Mould[2], Aine B. Henley[2], Alistair V. Nunn[2], Geoffrey W. Guy[3], E. Louise Thomas[2], Jameel M. Inal[4], Jimmy D. Bell[2], Sigrun Lange[5,6*]

[1] Cellular and Molecular Immunology Research Centre, School of Human Sciences, London Metropolitan University, London, UK.

[2] Research Centre for Optimal Health, Department of Life Sciences, University of Westminster, London, UK.

[3] GW Research, Sovereign House, Vision Park, Cambridge, CB24 9BZ, UK

[4] School of Life and Medical Sciences, University of Hertfordshire, College Lane, Hatfield, UK

[5] Tissue Architecture and Regeneration Research Group, Department of Biomedical Sciences, University of Westminster, London, UK

[6] Department of Pharmacology, University College London School of Pharmacy, London, UK

*** Correspondence:** Dr. Sigrun Lange; S.Lange@westminster.ac.uk

Supplementary Data

1.1 Supplementary Figure 1

Supplementary Fig. 1. EMV subpopulations isolated from the three cancer cell lines are exosomes and MVs. This is shown in Figures A-D as following by: A-B) electron microscopy, with exosomes (A) corresponding to vesicles in the < 100 nm NTA peak, and MVs (B) corresponding to the 100-500 nm NTA peak (scale bars are indicated as 50 nm for A and 200 nm for B respectively); C) PS exposition, which is enriched in the MV population (11,000*g* fraction; 100-500 nm peak on NTA); D) Western blotting for CD63 expression (exosome marker), showing increased expression of CD63 in the 100,000*g* EMV fraction (<100 nm peak on NTA).

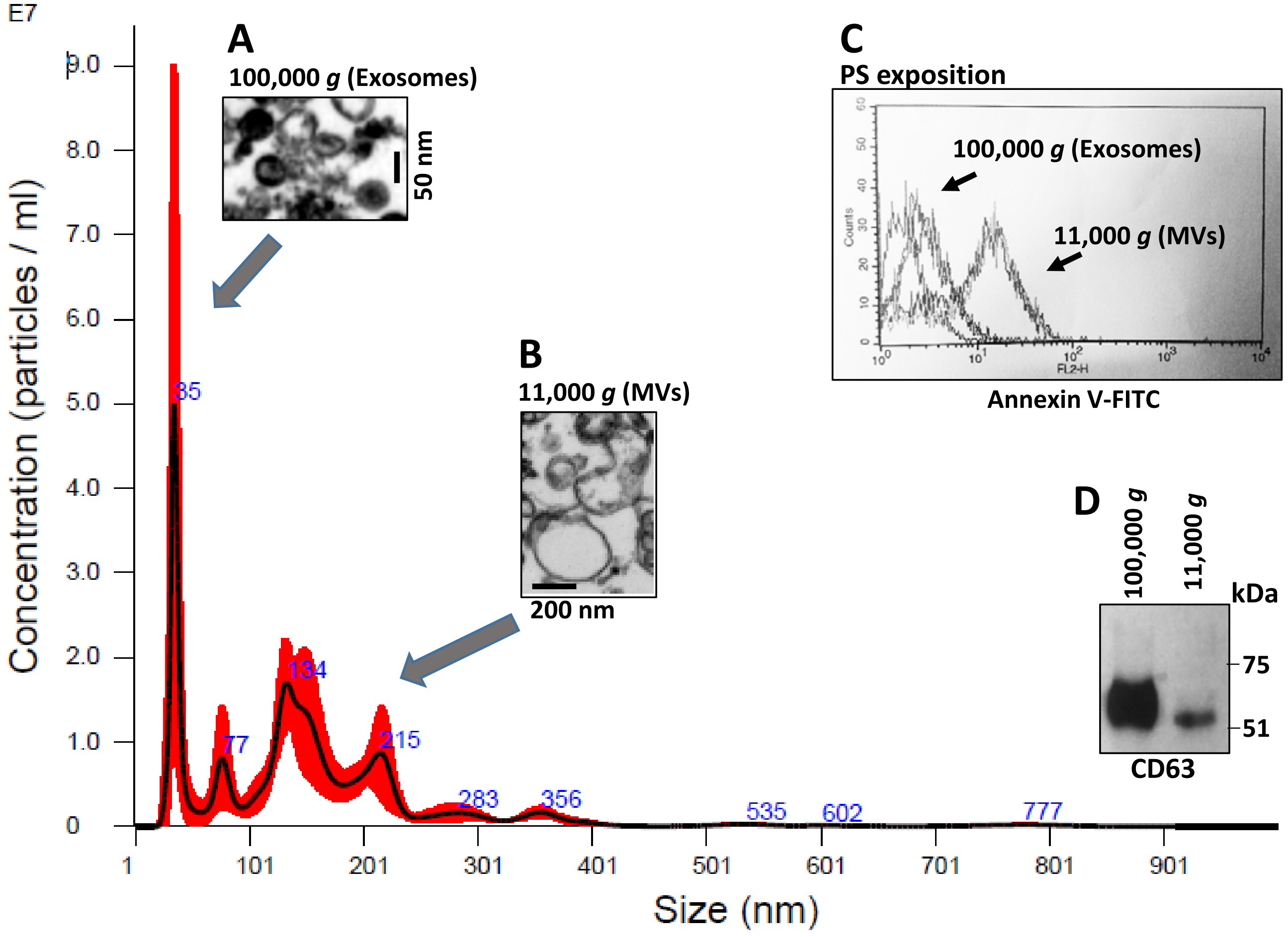

1.2 Supplementary Figure 2

Supplementary Fig. 2. HEPG2 and MDA-MB-231 cell viability following longer term (24 h) CBD treatment. Longer term (24 h) treatment effects of CBD on cancer cell viability was assessed for HEPG-2 and MDA-MB-231, showing dose-depended reduction in cell viability compared to control DMSO treated cells. In HEPG2 cells, 1 µM CBD resulted in a 38.8% decrease in cell viability, and 5 µM CBD in a 47.2% decrease in cell viability compared to DMSO treated control cells. In MDA-MB-231 cells, 1 µM CBD resulted in a 12.9% decrease in cell viability and 5 µM CBD in a 35.8% decrease in cell viability compared to DMSO treated control cells (*p<0.05, **p<0.01, ***p ≤0.001).

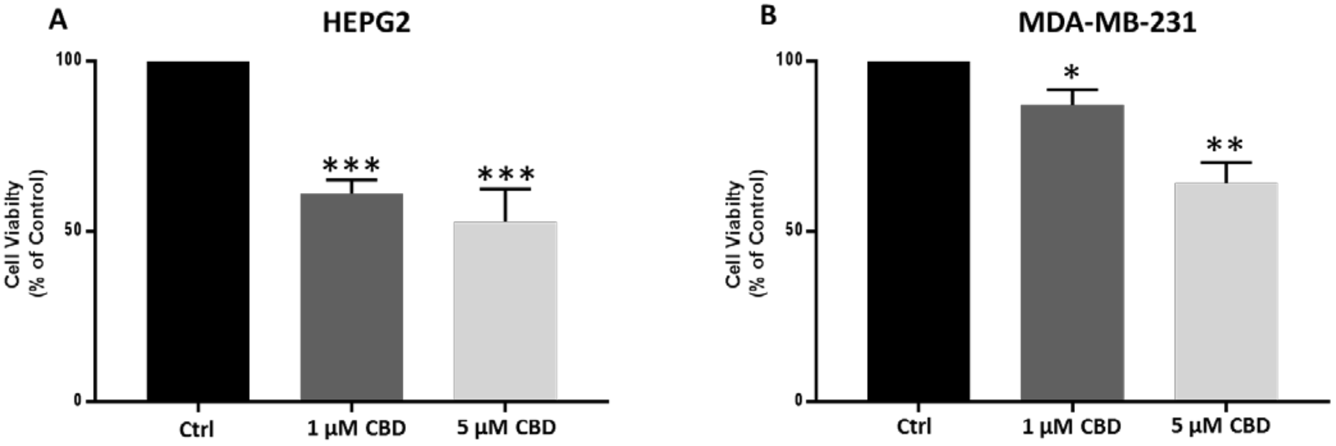

1.3 Supplementary Figure 3

Supplementary Fig. 3. EMV profiles of PC3, HEPG2 and MDA-MB-231 cancer cells. A) A range in the total amount of EMVs (0-900 nm) released was observed, as well as differences in the proportions of exosomes (<100 nm) and microvesicles (MVs; 100-900 nm) released from untreated cells (absence of CBD, Cl-amidine or DMSO). While PC3 cells released the highest amount of EMVs and similar proportions of exosomes and MVs, both HEPG2 and MDA-MB-231 released a higher proportion of MVs versus exosomes. B) In addition, a range in the proportional release of the two MV subsets at 100-200 nm and 201-500 nm are observed between the three cell lines, particularly regarding the 201-500 nm subset which is proportionally highest in HEPG2 compared to PC3 and MDA-MB-231 cells.

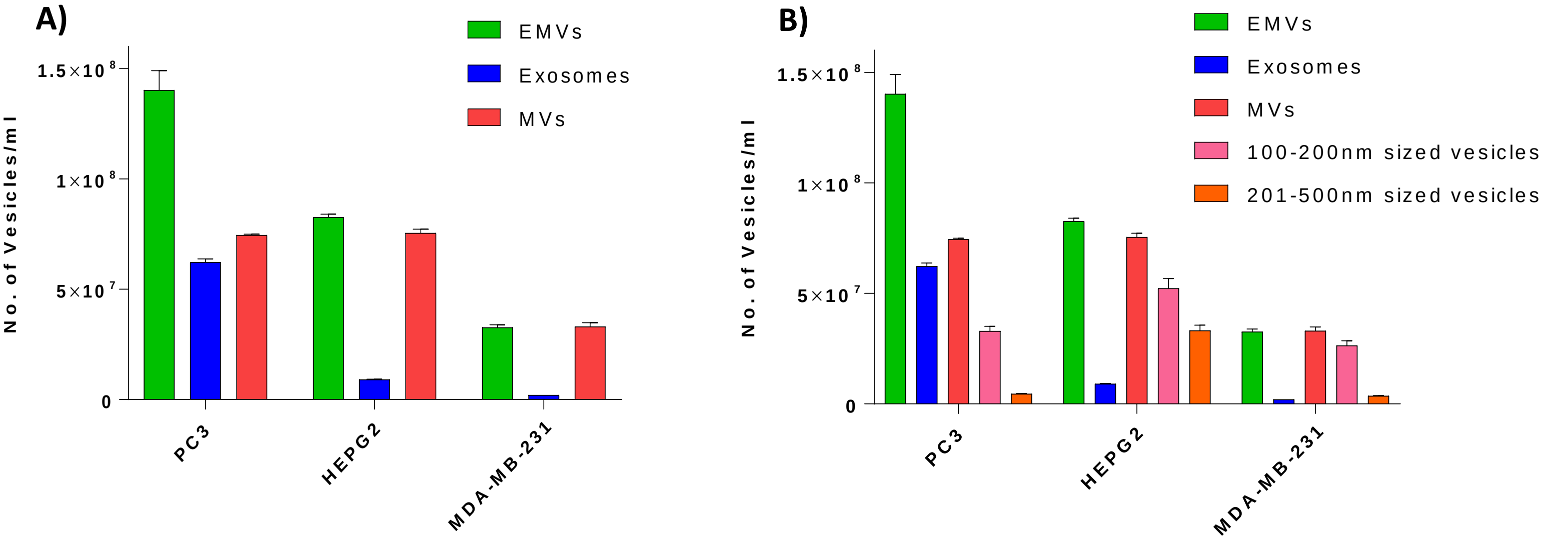

1.4 Supplementary Figure 4

Supplementary Fig. 3. Nanosight Tracking Analysis (NTA) of EMVs released from HEPG2 cancer cells in the presence of CBD, Cl-amidine and combinatory application of CBD and Cl-amidine. Representative plots from Nanosight tracking analysis show the concentration of total vesicles (0–900 nm in diameter) released from HEPG2 cancer cells. Vesicles outside this size range were excluded to avoid including larger vesicles such as MV aggregates or apoptotic bodies. The peak at 201-401 nm (as indicated by block arrows) observed in non-treated control cells (A) is notably reduced in CBD treated HEPG2 cells (B). This is also observed in combinatory treatment using Cl-amidine and CBD combined (D), while in the Cl-amidine treated cells the peak is present (C). The experiment was repeated three times in total with 5 readings performed for each sample for an average result per sample (error bars ± SEM, indicated in red).

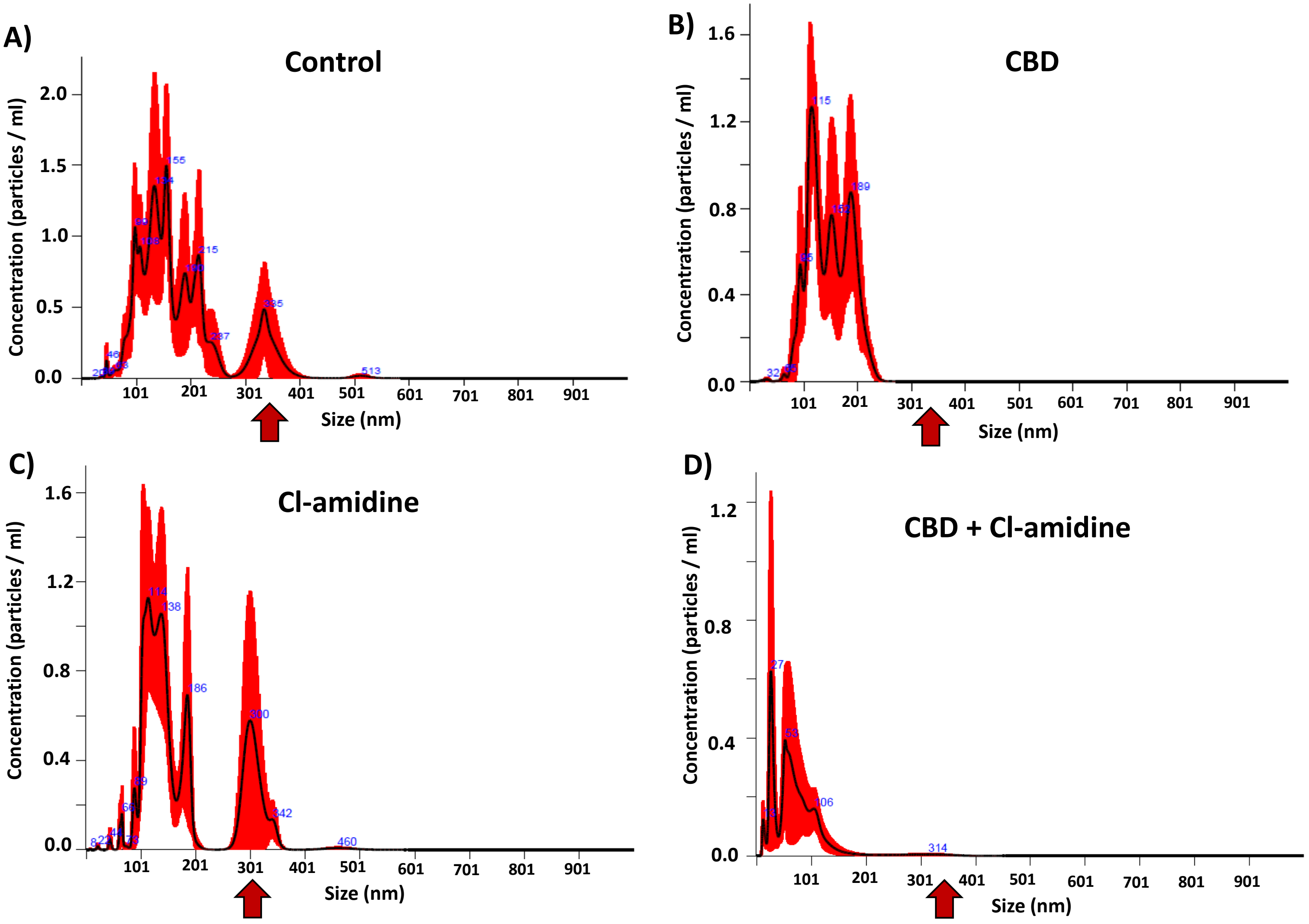

1.5 Supplementary Figure 5

Supplementary Fig. 5. ROS levels increase following 1 h CBD treatment in MDA-MB-231 cancer cells. MDA-MB-231 cells were treated with CBD for 1 h. ROS levels were measured after incubation using a 2′,7′-Dichlorofluorescin diacetate (DCFDA) assay according to the manufacturer's instructions (Abcam, UK). The amount of ROS was detected on a fluorescent plate read (FLUOstar Omega, BMG Labtech, UK) with an excitation of 495 nm and emission of 529 nm. The optical density was background subtracted and measured as a percentage of untreated cells after being normalized to cell count. This was carried out three times for experimental replication, with technical replicates of 5 wells per plate per treatment. Cancer cells showed a significant increase in ROS levels with increasing CBD; 1 µM CBD (1.30 ± 0.078 A.U.) and 5 µM CBD (1.84 ± 0.149 A.U.). Data shown is repeated 3 times with 5 replicates per plate and is normalised to cell count. Dashed line represents untreated levels. Data is represented as mean ± SEM; **p<0.01, ***p<0.001 versus control treated cells.

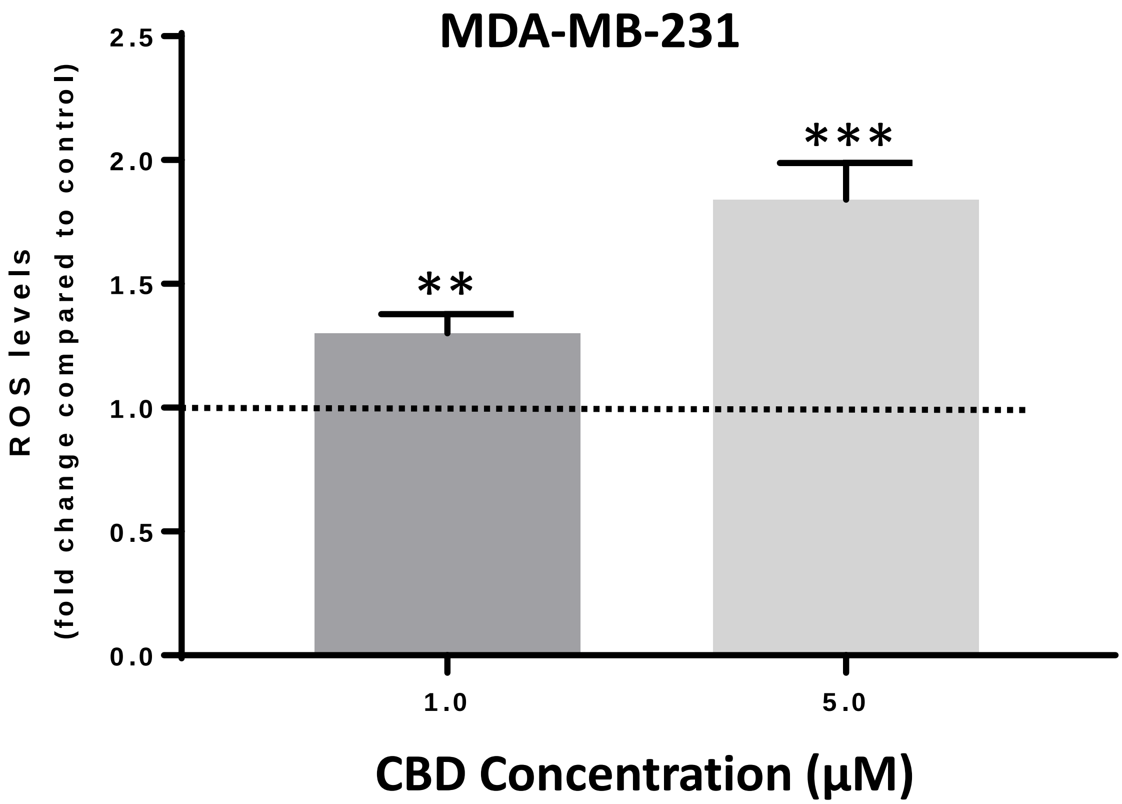

Supplement: Supplementary file 1 [file Data_Sheet_1.pdf]
